# Correlations between Clinical Judgement and Learning Style Preferences of Nursing Students in the Simulation Room

**DOI:** 10.5539/gjhs.v8n6p1

**Published:** 2015-09-28

**Authors:** Karin Hallin, Marie Häggström, Britt Bäckström, Lisbeth Porskrog Kristiansen

**Affiliations:** 1 Department of Nursing, Mid Sweden University, Sweden

**Keywords:** clinical skills, High Fidelity Simulation (HFS), Lasater Clinical Judgement Rubric (LCJR), learning skills, Nursing education, Productivity Environmental Preference Survey (PEPS), teamwork, videotaped scenarios

## Abstract

**Background:**

Health care educators account for variables affecting patient safety and are responsible for developing the highly complex process of education planning. Clinical judgement is a multidimensional process, which may be affected by learning styles. The aim was to explore three specific hypotheses to test correlations between nursing students’ team achievements in clinical judgement and emotional, sociological and physiological learning style preferences.

**Methods:**

A descriptive cross-sectional study was conducted with Swedish university nursing students in 2012-2013. Convenience sampling was used with 60 teams with 173 nursing students in the final semester of a three-year Bachelor of Science in nursing programme. Data collection included questionnaires of personal characteristics, learning style preferences, determined by the Dunn and Dunn Productivity Environmental Preference Survey, and videotaped complex nursing simulation scenarios. Comparison with Lasater Clinical Judgement Rubric and Non-parametric analyses were performed.

**Results:**

Three significant correlations were found between the team achievements and the students’ learning style preferences: significant negative correlation with ‘Structure’ and ‘Kinesthetic’ at the individual level, and positive correlation with the ‘Tactile’ variable. No significant correlations with students’ ‘Motivation’, ‘Persistence’, ‘Wish to learn alone’ and ‘Wish for an authoritative person present’ were seen.

**Discussion and Conclusion:**

There were multiple complex interactions between the tested learning style preferences and the team achievements of clinical judgement in the simulation room, which provides important information for the becoming nurses. Several factors may have influenced the results that should be acknowledged when designing further research. We suggest conducting mixed methods to determine further relationships between team achievements, learning style preferences, cognitive learning outcomes and group processes.

## 1. Introduction

The literature suggests there are many variables that may affect learning, and in order to match teaching and learning strategies nursing educators search for mechanisms to improve the effectiveness and efficiency of nursing students with diverse learning styles. The simulation room is an excellent environment in which students are allowed to make clinical judgements in situations concerning life and death, but many variables can positively or negatively affect the effectiveness of the clinical judgement process. Much has been studied on how nursing students interpret, conclude and act in care situations, but the nature of the relationships between clinical judgement and learning style preferences in practice are uncharted. Innovative thinking and hypothesis tests are needed to develop teaching and learning processes.

## 2. Background

### 2.1 Theoretical Framework

Becoming a skilled practitioner as a nurse requires first and foremost knowledge, competence and patient-safe environments, but also opportunities for reflection, reflection-in-action and/or reflection-on-action (Schön, 1987). John Dewey’s (1964) philosophical and theoretical thoughts on education as integrated unities of theory and practice are summarized in the concept of ‘learning by doing’. Reasoning and problem-solving in practical situations are performed in open, unstructured and perhaps also ambiguous situations, which can in one way or another be difficult to understand, cope with or resolve.

Dewey argues that knowledge is based on active learning with problem solving and critical thinking, which is needed to achieve the ability to determine what is relevant in a particular context. The importance of communication and interaction between individuals and groups, as well as motivation and self-interest, are largely a matter of acquiring information, understanding and skills (Dewey, 1964; Marton & Booth, 2000; Säljö, 2005).

High fidelity patient simulation (HFS) is one of only a few learning strategies in nursing education where students can demonstrate skills in complex care situations without harming the patient (Dieckmann, 2009; Nehring & Lashley, 2010). Within such abstraction, it could be argued that one’s clinical judgement (clinical thinking status) might be influenced by learning activities and vice versa.

### 2.2 Clinical Judgement in High Fidelity Patient Simulation

In nursing research the term ‘clinical judgement’ is synonymous with critical thinking, decision making and clinical reasoning (Tanner, 2006). Clinical judgement is one of the utmost essential skills for nurses to master in order to achieve the quality of nursing required. Tanner’s Clinical Judgement Model (CJM), with the four dimensions of *noticing, interpreting, responding* and *reflecting* describes nurses’ thinking and decision processes when they face complex care situations. In order to serve as a guide in the identification and analysis of nursing students’ skills and behaviour and to help students to diagnose failures, Lasater (2007) has developed the Clinical Judgement Rubric (LCJR) tool, on which students can be assessed as a meaningful whole. The evidence-based LCJR is like a checklist that indicates students’ development as being at the *beginning*, *developing*, *accomplished*, or *exemplary* level. The model and the rubric are extremely useful with computer-controlled human patient simulators in realistic patient-safe environments, and the students develop good clinical judgement skills (Dillard et al., 2009; Yuan, Williams, & Man, 2014).

Clinical judgement is a multidimensional process which is influenced by multiple sources, and probably also by learning style preferences because a person’s learning style is one of several important variables affecting learning outcomes (cf. Beischel, 2011; Shinnick & Woo, 2015). A systematic review indicated significant relationships between baccalaureate Bachelor nursing students’ critical thinking and learning styles. However, it should be noted that the six prospective and descriptive correlation studies published during 1994-2012 applied Kolb’s learning model as well as that of Felder and Silverman, but none applied the Dunn and Dunn Learning Style Model (Andreou, Papastavrou, & Merkouris, 2014).

### 2.3 Learning Styles

Learning style theories assume that all human beings learn in different ways and at different levels, which requires the ability to reflect on how students adapt teaching methods to their needs. This area of education is comprehensive and addresses students at both individual and group level, but also affects organizations as a whole (Beischel, 2011; Wheelan, 2009). It is apparent that the less academically successful the student, the more important it is to accommodate learning style preferences, and the stronger the preference, the more important it is to provide compatible strategies (Griggs et al., 1994). Researchers have identified a lot of areas that may influence students’ learning, one of which is the Dunn and Dunn Learning Style Model.

The Dunn and Dunn Learning Style Model, with its questionnaire, the ‘Productivity Environmental Preference Survey’ (PEPS) focuses on elements that are crucial for learning new and difficult information, but should not be confused with psychological models and tests. The model is built on fifty years of worldwide quantitative and qualitative research, which indicates that the model and the PEPS are very applicable to direct learning situations and the education of health professionals (Dunn & Griggs, 1998; 2007).

The PEPS focuses on 20 variables and yields information concerning the following:

- Environmental preferences: ‘Sound’, ‘Light’, ‘Temperature’, and ‘Furniture design’,
- Emotional preferences: ‘Motivation’, ‘Adjustability’, ‘Persistence’, the need for externally imposed ‘Structure’ or the opportunity to do things independently,
- Sociological preferences: ‘Authoritative person present’, ‘Variation/several ways’, ‘Learning alone, in pairs or as a part of a team’,
- Physiological preferences: perceptual strengths such as ‘Auditory’, ‘Visual’, ‘Tactile” or ‘Kinesthetic’, ‘Time-of-day energy levels’, need for ‘Intake’ and/or ‘Mobility’,

For example, the PEPS reveals individuals’ needs for either learning alone, with peers or with a teacher/supervisor. Tactile learners prefer hands-on learning, meaning that individuals “have active hands”, and let the hands move, draw and write during learning. Kinesthetic learners prefer the learning-by-doing approach and learn best through practical sessions, case studies or computer simulation where emotions are involved (Beischel, 2011; Hedin, 2006).

It should be noted that not all of the 20 elements of the PEPS need to be met for successful learning (Dunn & Griggs, 1998; 2007). The questions in this study do not pertain to talents, personalities and attitudes, but focus on learning preferences, clinical judgement and what is perceived to be difficult and new in high fidelity patient simulation.

### 2.4 Rationale

It is only when nursing students have the requirement to think like a nurse and to accurately notice, interpret, and respond to complex patient situations individually and/or in teams, that they can be considered fully trained. When it comes to measuring the ability to conduct proper clinical judgements, HFS is a very adequate methodology for nursing students, and as educators we need to be considering the best probable learning outcomes. Much is studied about HFS, learning styles and clinical judgment skills independently, but knowledge about the relationships between these variables is limited. Rethinking and new kind of hypotheses are needed in order to develop educational methods. In an earlier study with the same sample and a similar design to this study, the results showed some significant positive correlations (Hallin et al., 2015), for instance, being observers and having HFS experience prior to nursing education, while personal characteristics such as gender, age, work experience and scenario circumstances were of secondary or no importance. Therefore, this study will focus on the emotional, sociological and physiological preferences as being considered the most relevant, because the simulation environment is vivid and unstructured, without authoritative teachers to lead, and the students in teams are expected to make decisions independently and in interaction. The aim was to explore correlations between final semester pre-licensure Bachelor nursing students’ clinical judgement and their learning styles, and though this is not an experimental study, we specifically want to test the following hypotheses:

- There will be positive significant correlations between team achievements of clinical judgement and students’ “Motivation” and “Persistence”.
- There will be negative significant correlations between team achievements of clinical judgement and students’ “Wish for an externally imposed structure”, “Learning alone” and “Wish for an authoritative person present”.
- There will be positive significant correlations between team achievements of clinical judgement and students’ “Tactile” preference (hands involved), and “Kinesthetic” preference (learning by doing, physically and/or emotionally involved).

## 3. Method

### 3.1 Design

A descriptive cross-sectional quantitative study was conducted with nursing students at a Swedish university in 2012-2013. Two underlying models shaped the study: the Clinical Judgement Model (CJM) with aspects of how nurses think and act in complex situations (Tanner, 2006), and the Dunn and Dunn Learning Style Model (Dunn, Dunn, & Price, 2000), which primarily focuses on the acquisition of new and difficult information. Teamwork and team size were chosen based on the complex situations that the students would meet as graduated nurses in about ten weeks’ time (Wheelan, 2009). The study is part of a larger quasi-experimental project entitled ‘Values and effects of high fidelity simulation for the development of clinical judgement’. Pilot studies (unpublished) were carried out in Spring 2012 which confirmed that the study design was relevant for the coherent and individual studies in terms of participants, measurements, data collection and choice of analysis methods.

### 3.2 Participants

Convenience sampling was used and the students were recruited from five groups from two campuses at one university. The participants, 173 of 241 nursing students (138 female and 35 male), were in the final semester of a three-year Bachelor of Sciences Nursing programme and all studied according to the same curriculum. In current study curriculum they were expected to integrate all theoretical and practical knowledge achieved during the whole education period, and they should function as autonomous registered nurses within a few weeks. The loss of 68 students was due to two reasons: following the video recording one of the members refused further participation and the whole team was excluded, and some video footage was of poor quality.

As shown in Table 1, the majority of the participants were female students (79.8%), aged 21-30 (80.5%), most without an assistant nursing degree (80.3%), and without previous HFS experience (89.6%). Healthcare work experience before nursing education included no experience (19.0%), less than 1 year (20.1%), 1-5 years (45.4%) and more than 5 years (15.5%).

**Table 1:** Nursing Students’ Characteristics (*n* = 173)

Variables		%	*n*	Min-Max	Mean	Median	Mode	SD
Gender	Female	79.8	138					
	Male	20.2	35					
Age	Years		173	21-48	26.64	25.5	22	5.05
Healthcare work experience before nursing education	(months)		173	0-240	31.47	18.0	0	47.10
Assistant nurse graduation before nursing education	Yes	19.7	34					
	No	80.3	139					
HFS experience outside nursing education	Yes	9.2	16					
	No	89.6	155					
	Missing	1.2	2					

These students had previously participated in a study (Hallin, 2014) which showed no significant correlations between the characteristics of the five student semester groups at the two campuses of the university. The results showed those student teams to be homogeneous.

During their nursing education the students had used various teaching methods, such as lectures, tutorials, computer-based methods, individual and group work, case studies and practical exercises with low fidelity patient simulation, but had not spent any time on HFS.

### 3.3 Measurements and Data Collection

Aside from videotaped simulation scenarios, the data collection included two questionnaires aimed at identifying personal characteristics and learning style preferences. The Swedish translation of the PEPS, the Productivity Environmental Preference Survey for adults (Dunn, Dunn, & Price, 2000), consists of 100 questions relating to 20 distinct learning style elements concerning environmental, emotional, sociological and physiological preferences, each with a five-item Likert scale ranging from 1 (never) to 5 (always). The reliability and validity of the PEPS are considered good as the questionnaire has been widely used in various professions including healthcare (Griggs et al., 1994). For each element the reliability coefficient typically falls into the .75 to .88 range (Dunn et al., 1995), and the construct validity evidence has been proven in a variety of high-quality international research works (Dunn et al., 1995; Nelson et al., 1993).

Answering the questionnaires took approximately 30 minutes and was done in connection with a lecture before the HFS scenarios. At the time of data collection the students had completed all courses of the nursing programme except the final clinical course (ten weeks).

### 3.4 Simulation Scenarios

Educators at a clinical training centre created HFS scenarios in an experimental hospital environment to exercise clinical judgement skills. The students were divided into 60 teams consisting of two, three or four students without regard for personal characteristics. In order to making the students familiar with both a medical and a surgical care situation two scenarios unknown to the students were given to each team: one patient with septic shock and one patient with haematemesis in which the team were either actors or observers. Between each scenario the students changed roles.

The preparation, implementation and monitoring of the HFS scenarios, as well the measurement and treatment of the airway, breathing, circulation, disability, exposure (A, B, C, D, E) parameters followed proven recommendations (Dieckmann, 2009; Nehring & Lashley, 2010). Before the simulation the students were informed of the learning outcomes and were given the opportunity to become familiar with the simulator and the care environment. They were also told to think aloud and work and collaborate as a nursing team.

An operator in an adjacent room controlled the simulator and acted as the voice of the patient. A trained facilitator observed the students’ behaviour, answered questions about variables that could not be simulated and sought medical advice when required. Each scenario lasted about 10-15 minutes. Before changing roles the actors and observers were debriefed of the process under the guidance of the facilitator.

### 3.5 Analysis

Quantitative analysis methods were used in this study. The nursing students’ characteristics, the demographic data, were analysed using descriptive statistics while the hypotheses were tested using non parametric rank correlation.

The students’ team actions in the HFS scenarios were analysed using the Lasater Clinical Judgement Rubric (LCJR) matrix (Lasater, 2007), based on Tanner’s Clinical Judgement Model (Tanner, 2006). The LCJR was developed in order to analyse and score students’ ability to notice, interpret and respond in complex care situations in grades of four development stages: beginning, developing, accomplished and exemplary. Nine dimensions in total were analysed with 1 to 4 points awarded for each, which meant that each student team was able to achieve a maximum of 36 points. Reflection, the fourth construct in Tanner’s model, was excluded as it was carried out individually in written form as part of this study.

The scenario analyses were carried out by four senior lecturers and one lecturer in collaboration. They ensured inter-rater reliability with a result of 0.86. The values were calculated at group level and were used for inferential statistics. According to several references the LCJR is a well-designed measure of clinical judgement skills (Shipman et al., 2012) with good validity and reliability for use in HFS (Adamson et al., 2012). The LCJR was translated from English to Swedish and back to English (Polit & Beck, 2012) with great compliance (Kristiansen et al., 2015).

The responses to the PEPS were computer processed with the results of each student’s preferred learning style traits based on the 20 variables. The individual profiles showed an average for each question on a 60-point scale and marked the student’s values as low (average 20-40), flexible (average 41-60) and high (average 61-80).

For further processing the Statistical Package for Social Sciences (SPSS Inc., Chicago, IL, USA), version 20 for Windows was used and the level of significance was defined as *p* < 0.05 (two-tailed).

Associations between team achievements of clinical judgement (dependent variable with skewed quota scale) and learning style preferences (independent variables with ordinal scales) were performed with Spearman’s rho correlation coefficient in two ways: first with bivariate correlations at the individual level of 173 students, and then with covariance at the team level of 60 teams. We have worked driven by hypotheses, although we have tested all the learning style elements and team achievements for correlation significance.

### 3.6 Ethical Considerations

This study, which is part of a larger project, was approved without any objections by the University Research Ethics Committee (Ref. No. 2012/499). The students signed their informed consent for participation and the study was conducted according to applicable ethical principles with a confidential process. There was no dependent relationship between the students and the researchers.

## 4. Results

The recorded HFS scenarios were analysed by the Lasater Clinical Judgement Rubric (LCJR), and the teams demonstrated abilities in line with the stages of ‘Beginning’, ‘Developing’ or ‘Accomplished’ (Table 2).

**Table 2:** Nursing students’ team achievements measured by the lasater clinical judgement rubric

Achievements of 60 Team-points	teams n teams	%	Stages
9	5	8.33	Beginning
10-15	15	25.00	Beginning-Developing
16-20	12	20.00	Developing
21-25	17	28.34	Developing-Accomplished
26-30	11	18.33	Accomplished
31-35	0	0	Accomplished-Exemplary
36	0	0	Exemplary

Total	60	100	

*Note*. Possible points = 36, Min-Max= 9-30; Mean=19.0; Median= 19.5; SD=6.25.

When analysing the learning style preferences we used the PEPS scale. In Table 3, it is shown that the majority of the students demonstrated figures between 41 and 60, the middle and flexible range without strong preferences. Furthermore, the results showed that only a few of the students scored below 40 and several demonstrated scores above 60 on each of the learning style elements.

**Table 3:** Learning style preferences: Distribution of low, flexible, and high preference scores for 173 nursing students

Elements ^†^	Low Scale 20-40			Flexible Scale 41-60		High Scale 61-80			Total
		*n*	%	*n*	%		*n*	%	*n*
**Environmental preferences**									
Sound	Prefers quiet	1	0.6	143	82.7	Prefers noise	29	16.8	173
Light	Prefers dim	12	6.9	145	83.8	Prefers bright	16	9.2	173
Temperature	Prefers cool	2	11.6	131	75.7	Prefers warm	22	12.7	173
Furniture design	Prefers informal	15	8.7	125	72.3	Prefers formal	33	19.1	173
**Emotional preferences**									
Motivation	Low own drive	5	2.9	140	80.9	High own drive	28	16.2	173
Persistence	Not preserving	0	0.0	115	66.5	High preserving	58	33.5	
Adjustability	Does not prefer adjustment	20	11.6	138	79.8	Prefers adjustment	15	8.7	173
Structure	Does not prefer	0	0.0	38	22.0	Prefers	135	78.0	173
**Sociological preferences**									
Authoritative person	Does not want present	1	0.6	101	58.4	Wants present	71	41.0	173
Several Ways	Does not prefer	28	16.2	140	80.9	Prefers variety	5	2.9	173
Alone/Peers	Prefers alone	7	4.0	48	27.7	Prefers with peers	118	68.2	173
**Physiological preferences**									
Auditory	Does not prefer	7	4.0	109	63.0	Prefers	57	32.9	173
Visual	Does not prefer	23	13.3	137	79.2	Prefers	13	7.5	173
Tactile	Does not prefer	6	3.5	117	67.6	Prefers	50	28.9	173
Kinesthetic	Does not prefer	1	0.6	126	72.8	Prefers	46	26.6	173
Morning	Does not prefer	28	16.2	116	67.1	Prefers	29	16.8	173
Late morning	Does not prefer	32	18.5	107	61.8	Prefers	34	19.7	173
Afternoon	Does not prefer	20	11.6	101	58.4	Prefers	52	30.1	173
Intake	Does not prefer	14	8.1	122	70.5	Prefers	37	21.4	173
Mobility	Does not prefer	6	3.5	130	75.1	Prefers	37	21.4	173

† Items with Likert scales: 1 (never) to 5 (always).

As shown in Table 4, the correlations between team achievements of clinical judgement and learning style preferences regarding the elements of the hypotheses, ‘Motivation’, ‘Persistent’, ‘Structure’, ‘Authoritative person present’, ‘Alone/Peers’, ‘Tactile’ and ‘Kinesthetic’ were calculated. Three significant correlations were found. The results showed a negative correlation between the team achievements and the students’ preference of ‘Structure’, a positive correlation between the team achievements and the preference of ‘Tactile’, and a negative correlation between the team achievements and the preference of ‘Kinesthetic’ at the individual level, but which was not applicable to covariance at the team level (Table 4).

**Table 4:** Correlations between team achievements of clinical judgement and learning style preferences at the individual level (173 nursing students) and covariance at the team level (60 teams)

Learning style preferences Scale 20-80	Team achievements of clinical judgement			

	Individual level Spearman’s rho correlation coefficient		Covariance at team level Spearman’s rho correlation coefficient	
	
	Correlation Coefficient	*p*-value Asymp. Sig. (two-tailed)	Correlation Coefficient	*p*-value Asymp. Sig. (two-tailed)
**Emotional preferences**				
Motivation	0.018	0.813	0.023	0.859
Persistence	0.058	0.446	0.128	0.328
Structure	**-0.242**	**0.001**	**-0.423**	**0.001**
**Sociological preferences**				
Authoritative person present	-0.061	0.425	-0.064	0.629
Alone/Peers	-0.023	0.759	-0.106	0.420
**Physiological preferences**				
Tactile	**0.166**	**0.029**	**0.285**	**0.027**
Kinesthetic	**-0.154**	**0.043**	-0.188	0.149

*Note*. Points of a possible 36: min = 9; max = 30.

Further analysis of the learning style preferences in Table 3 showed no significant correlations with the students’ team achievements of clinical judgement (*p* = .32 - .92).

## 5. Discussion

### 5.1 Summary of Results

The aim was to explore the correlations between pre-licensure nursing students’ clinical judgement and their learning styles. Three hypotheses were tested from the results of the LCJR for clinical judgement and the PEPS for learning style preference. Although three significant correlations were found, only parts of the second and third hypotheses were confirmed. Approximately a third (33%) of the student teams achieved low scores in clinical judgement and demonstrated abilities in line with the level ‘Beginning/Developing’, while the majority of the teams (67%) achieved 16 points or more. Although only 18% achieved more than 26 points on a scale with a maximum of 36 points, a few teams demonstrated abilities equivalent to the level ‘Accomplished’. None of the 60 teams demonstrated abilities in line with the ‘Exemplary’ level phase. The majority of the students were found to be in the middle and flexible range of the PEPS scale, which means that they were without strong learning style preferences. Only a small number of the students scored below 40 points (does not prefer), but several students demonstrated scores above 60 (prefers) on each of the learning style elements, indicating that they would benefit from special accommodation to their learning style preferences.

### 5.2 Discussion of Results

#### 5.2.1 Positive Correlations between Team Achievements and ‘Motivation’ and ‘Persistence’

This hypothesis was rejected as the results showed no significant correlations between team achievements of clinical judgement and the students’ ‘Motivation’, and ‘Persistence’. We expected significant positive correlations as motivation and persistence especially are central and necessary for effective outcomes in education (Dewey, 1964). A reasonable explanation may be that the majority of the students were ‘flexible’ in their motivation, and seemingly lacked adequate nursing knowledge to manage clinical judgement in teams. To achieve good learning in the ‘flexible’ region, the students are dependent on a strong interest. If not interested, they learn superficially on external motivation and only in short-term memory (Dunn & Griggs, 2007). To satisfy those students two, three or four learning style preferences need to be involved in deep learning (AlKhasawneh, 2013; Dunn & Griggs, 2007). When discussing motivation and persistence, the distinction between internal and external motivation is of great importance. The reason for taking part is to find inner joy in the work or hope for future reward. We know from an inner motivational drive that the simulation environment favoured the students’ capacity to solve problems independently (Botma, 2014).

Most likely, there were other factors that influenced the relationship between ‘Motivation’/‘Persistence’ and team achievements of clinical judgement; the team members may even have experienced negative feelings or decreased cognitive learning following group work and low/middle fidelity simulation (cf. Beischel, 2011). Due to the fact that for most of the students it was their first time in HFS, nervousness, anxiety and weak team development might have been possible explanations. According to Beischel (2011), within balanced boundaries, the readier students are to learn and prepare for simulation the less anxiety they experience. We assumed that the participating nursing students would regard the HFS scenarios as an immersive and highly motivating method bringing theory into practice (cf. Botma, 2014), although this seems to be of minor importance in our study.

Another explanation for the rejected hypothesis might be that the students actually misjudged themselves. In fact, 2.9% and 0% of the students scored low levels in the learning style elements ‘Motivation’ and ‘Persistence’, respectively. Therefore, a rhetorically relevant question would be: to what extent were the team members motivated and persistent in studying nursing? Actually, few teams demonstrated abilities equivalent to the level of ‘Accomplished’. An earlier study with the same sample showed that the majority of the teams needed to be performing highly theoretically in order to attain high team achievements in clinical judgement (Hallin et al., 2015). We suggest conducting further studies to test our hypothesis.

#### 5.2.2 Negative Correlations between Team Achievements and ‘Structure’, ‘Learning Alone’ and ‘Authoritative Person Present’

The hypothesis was confirmed for the first part and rejected for the second. There was a significant negative correlation between the team achievements and externally imposed ‘Structure’. All the students preferred structure, of which 78% preferred a high level of structure. Because the scenario arrangements in HFS environments are characterized by self-directed teamwork with a low level of structure, we saw a significant negative correlation as logical. The students preferred to act on concreteness, clear directives and guidelines, which can only partially be fulfilled in an HFS exercise. Nevertheless, using the structure of observation and a role model will give positive correlations in clinical judgement. Lasater et al. (2014) exhibited an expert nurse role model on a video caring for a patient similar to the HFS patient; the treatment group watched the video and it was found that an expert role model can have an impact on nursing students’ self-confidence and development of clinical judgement. Being observers and/or having HFS experience before nursing education can provide significant positive outcomes (Hallin et al., 2015). How role models correlate and impact on the PEPS element ‘Structure’, the clinical judgement of the LCJR, and complex HFS care situations would be interesting to study further.

There were no significant correlations between the team achievements in clinical judgement and the students’ ‘Wish to learn alone’ or ‘Wish for an authoritative person present’. We expected negative correlations as the simulations are performed in teams and without a facilitator present guiding the parts of the scenario. In fact, of the students’ heterogeneous learning style preferences just 41% had high preference for learning together with peers, while 68% preferred an authoritative person to be present, compared with 78% who preferred high structure. But the question remains how the degree to which students’ different learning style preferences impact on learning and knowledge in teamwork. More studies are needed.

#### 5.2.3 Positive Correlations between Team Achievements and ‘Tactile’ and ‘Kinesthetic’

This hypothesis was also rejected. The results showed no significant correlations between team achievements and the ‘Tactile’ learning style preference (having active hands during learning), and a significant negative correlation at the individual level only with the ‘Kinesthetic’ variable (learning by doing, physically and/or emotionally involved). More than a quarter of the students had high preference for a tactile (29%) and kinesthetic (27%) learning style, while 68% and 73%, respectively, were flexible in their preferences. This can be compared with the study of James, D’Amore and Thomas (2011), using the VARK test (visual, aural, reading/writing and kinesthetic preferences), which found that first year nursing and midwifery students scored significantly high in the ‘Kinesthetic’ preference. We expected significant positive correlations as tactile and kinesthetic students learn best through practical sessions and case studies where hands and emotions are involved, but we do not have any reliable suggestion as to how to understand and interpret this aspect of the results. HFS scenarios, with their hands-on approach, ought to fit these students like a glove. In contrast, the results of Beischel (2011) indicated that strong hands-on learning styles can be negatively related to cognitive outcomes. This could be a possible explanation for our result; the teams were more or less low achievers, but we do not know the students’ cognitive outcomes. More studies are needed to investigate how physiological preferences such as tactile and kinesthetic preferences correlate to individual and team achievements in clinical judgement.

In total, three significant correlations were found between the pre-licensure nursing students’ team achievements of clinical judgement and their learning style preferences, and just one part of the second and one part of the third hypotheses were confirmed. So, why was there such a poor match between the logically formulated hypotheses and the displayed results? We have speculated as to whether the context of the HFS scenarios was more significant than the PEPS learning style preferences, but such thoughts can be more or less dismissed, as Shinnick and Woo (2015) have confirmed in an experimental study that HFS is an effective teaching method for pre-licensure nursing students with most types of Kolb’s learning styles. Similar success between learning and the use of HFS has also been detected by Botma (2014). Therefore, what other factors may have influenced the correlations between learning style preferences and clinical judgement?

### 5.3 General Discussion of Results

Our confirmed and rejected hypotheses need further consideration in order to help teachers and students to connect learning, knowledge, and competence development to ensure high levels of nursing education and patient safety. It is evident that there are multiple complex interactions between learning style preferences and team achievements in the simulation room. Dewey (1964) never considers learning processes as being distinct from the context, and Dunn, Dunn and Price (2000) argue that concrete action in reality occurs in the interaction between learning and cooperation with others. Emotional learning style preferences such as ‘Motivation’, ‘Responsibility’, ‘Conformity’, and ‘Persistence’ are needed for the opportunity to do things independently and in teams in unstructured environments without an authoritative person present (Dunn, Dunn, & Price, 2000).

Dewey (1964) coined the term ‘learning by doing’, an activity in which pedagogy, theory, practice, reflection and action are linked. In other words, learning becomes a matter of collecting experiences from different situations, not least from HFS, and uses the experiences as a basis for action strategies in like-new situations. Nevertheless, the less academically successful the students are, the more important it is to accommodate learning style preferences, and the stronger the preferences are, the more important it is to provide compatible strategies (Griggs et al., 1994). Obviously, if the students working alone or in teams do not achieve the expected knowledge and skills, patients’ safety is jeopardized. Cormier, Pickett-Hauber and Whyte (2010) found in simulated environments that low-performing nursing students observed many irrelevant cues. They verbalized more irrelevant information cues and failed to perform response actions directly related to the patient’s condition. High-performing nursing students were better at observing relevant cues, and were better able to recognize salient symptoms suggesting patient deterioration. Furthermore, found high-performing nursing students were able to forward-plan response actions that positively altered the physiological trajectory of the patient (Cormier et al., 2010). McRobert et al. (2013) saw similar outcomes in skilled and less skilled physicians in simulated medicine emergencies.

Aside from the importance of knowledge and skills, an interesting question is how the students impacted on each other in their teams. We know that emotions cause a wide range of effects on processes involving group work and thus also in clinical judgement (cf. Beischel, 2011), so how confident and how uncertain were the students in the HFS teams? Admittedly, the HFS situation was the students’ first experience of a more complex nursing situation, but at the same time those third-year university nursing students had taken part in a lot of teamwork during their education. They had discussed complex patient situations theoretically and used teamwork in low and middle fidelity simulations several times previously during their education. Besides, almost a third of their education is praxis-based and supervisor-led at hospitals or in nursing homes. In any case, students and nurses are expected to interact in different team configurations without regard for group processes, but according to Wheelan (2009) all groups undergo four development phases:

- Phase 1: Dependency and inclusion marked by high anxiety, uncertainty, and politeness
- Phase 2: Counter-dependency and fighting marked by conflict, power struggles, search for identity and definition of roles
- Phase 3: Trust and structure marked by more mature negotiation processes, team goals, organizational structure, procedures, roles and division of laboure
- Phase 4: Work and termination characterized by team members feeling comfortable with one another and the habitual sharing of information.

An important point to note is that if more than half of the members are replaced, the group will regress to Phase 1.

Supporting the results of Wheelan (2009), we have to deepen our understanding and take the development of the group processes into consideration when studying the students’ learning and clinical judgement skills. Something that might have had a negative influence on the students’ achievements is that the classes were large, and several students had left and joined classes based on individual course outlines. During their clinical education the students had worked in staff teams with constantly changing work schedules and changeable employment. None of our 60 teams demonstrated trust in the expertise of the group. Instead, the team members were more likely to be hesitant and concerned with issues such as being accepted and reducing uncertainty, and therefore tended to be defensive and defer to the team’s designated leader. Good benchmarks are that staff/groups/teams containing 3-8 members are more productive and more developmentally advanced than those with nine members or more (Wheelan, 2009). This is reinforced by staff members of high- performing units who perceive their teams as more structured and organized then staff members of lower-performing units (Wheelan, Burchill, & Tilin, 2003). According to Schön (1987) skilled practitioners and teams will access repertoires of images, interpretations and documents used in reflection-in-action and/or reflection-on-action, some taken over from professional communities, others based on the person’s own experiences.

### 5.4 Discussion of Method

The study had both strengths and limitations. HFS is an effective teaching and learning method (Kirkman, 2013; Shinnick & Woo, 2015), suitable for training and testing clinical judgement (Kelly, Hager, & Gallagher, 2014; Lasater, 2007; Tanner, 2006), and the instruments of LCJR (Adamson et al., 2012) and PEPS (Dunn et al., 1995; Griggs et al., 1994) are recognized as being valid and reliable. The researchers’ inter-rater reliability with the result of 0.86 is considered sufficient (Kristiansen et al., 2015). As shown in an earlier study with the same students, demographic factors such as gender, age, healthcare experience and possession of an assistant nursing degree would not have affected the outcomes of team achievements of clinical judgement (Hallin et al., 2015). Despite a loss of 28.2% (*n* = 68), which arose when a single student decided to not participate and the student’s entire team were excluded, a large number of nursing students (*n* = 173) from five semester groups at two campuses participated in 60 teams. However, as the data collection was done only once for each team at a single university with pre-licensure nursing students, and there is need for encouragement and ongoing practice in different HFS situations for long-term change and to maintain competence (Dillard et al., 2009), the results should be considered with some caution. Our intention was to formulate hypotheses in a pilot study and generate a basis for further research.

## 6. Implications and Conclusions

There were poor matches between the logically formulated hypotheses and the displayed results, and the rejected hypotheses surprised us researches, experienced Registered nurses, Nursing educators and Senior lecturers. The results indicated that it is no longer sustainable to assume that all adult students of a class learn in the same way. However, when our results were discussed in the light of other results, it clarified that students with dissimilar learning styles and clinical judgement abilities will in simulation situations be influenced by factors other than those teachers and students would normally expect. To improve nursing students’ learning results in order to adapt learning and teaching methods educators have to map the students’ learning styles tentatively two times during nursing education (Hallin, 2014). Variables in attention should be the preferences of Motivation, Persistence, Tactile and Kinesthetic, Wish for an externally imposed structure, learning alone/in pairs/in groups, and Wish for an authoritative person present. Based on the results the tuition should be discussed and confirmed between teachers and students, in HFS scenarios as a separate moment in the debriefing process. HFS is an attractive learning method to nursing faculty members, especially when the majority of complex care situations are virtually impossible for pre-licensure nursing students to experience and deal with in clinical environments, and when students in a nursing course are composed of learners with many different strengths and weaknesses. The task is to see and pay attention to each unique student in the group. We implement Dewey’s (1964) pedagogy that revolves around developing experience. To ensure patient safety and students’ pride in being a nurse, we also adopt Wheelan’s (2009) development and progression of group processes to reach phase 4. The role of teachers and educational managers is chiefly responsible for the highly complex process of education planning. The task is to take stock of the level of students’ knowledge and experience in clinical judgement, and to provide a unique setting in which to conduct learning and working individually and in teams. In doing this the teacher must also be willing to learn and to know a lot of learning style preferences. Dewey looks upon the relationship between teacher and pupil as a mutual one, and the importance of motivation and self-interest, communication, reflection and interaction between individuals and teams, which is largely a matter of acquiring information, understanding and skills (Dewey, 1964; Marton & Booth, 2000; Schön, 1987; Säljö, 2005). The PEPS and the LCJR provide a scholarly framework for enhancing students’ judgement for nursing practice and learning style preferences. A reasonable conclusion is that further correlation studies between learning style preferences and team achievements in clinical judgement should be conducted in student groups that have reached phases 3 and 4 in group development. A key point in the progressive education movement is that students need to have choice, meaning, purpose and motivation throughout the whole of their learning process (Dewey, 1964). Further, the students, who are just about to transform into a clinical active practitioner stage, i.e. Registered nurses, where personal responsibility and awareness is of great importance. We assume that they will benefit from knowledge of their own learning preferences in their future professional journey.
